# A Rare Case Diagnosed by Videothoracoscopic Lung Biopsy: Diffuse Pulmonary Meningotheliomatosis

**DOI:** 10.1155/2021/1990433

**Published:** 2021-08-28

**Authors:** Anton Dzian, Marek Malík, Ľuboš Hamada, Jozef Mičák, Ivana Gregorová, Gabriela Košturiaková

**Affiliations:** ^1^Department of Thoracic Surgery, Jessenius Faculty of Medicine in Martin, Comenius University in Bratislava, University Hospital in Martin, Kollárova 2, 036 59 Martin, Slovakia; ^2^Department of Pathological Anatomy, Jessenius Faculty of Medicine in Martin, Comenius University in Bratislava, University Hospital in Martin, Kollárova 2, 036 59 Martin, Slovakia; ^3^Specialised Hospital of St Zoerardus Zobor, Kláštorská 134, 949 88 Nitra-Zobor, Slovakia

## Abstract

Diffuse pulmonary meningotheliomatosis (DPM) is reported as a diffuse parenchymal lung disease characterized by disseminating small asymptomatic nodules. These lesions are often detected incidentally as microscopic findings in lung specimens or autopsies examined by a pathologist. We report a case of a 60-year-old male asymptomatic patient presenting with multiple bilateral pulmonary nodules on high-resolution computed tomography and diagnosed by videothoracoscopic surgery. Differential diagnosis of patients presenting with diffuse indeterminate nodules is very important. Definitive diagnosis of DPM requires histopathology and most often videothoracoscopic lung biopsy.

## 1. Introduction

Diffuse pulmonary meningotheliomatosis (DPM) is a rare disease characterized by the presence of diffuse benign small nodules of uncertain clinical significance. These nodules consist of epithelioid cells, which have an origin in the mesenchymal meningothelial cell line. These lesions are often detected incidentally as microscopic findings in lung specimens or autopsies examined by pathologist. We report a case of a patient with this rare finding in videothoracoscopic lung biopsy.

## 2. Case Presentation

**Figure 1:**
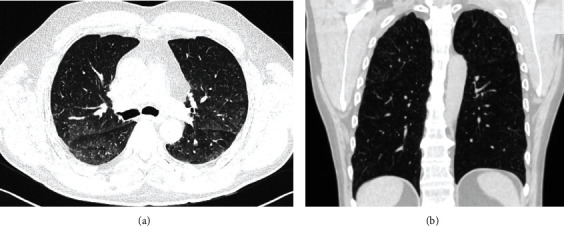
High-resolution computed tomography (HRCT) images (a, b) demonstrate diffuse, multiple nodular lesions in the parenchyma of both lungs.

**Figure 2:**
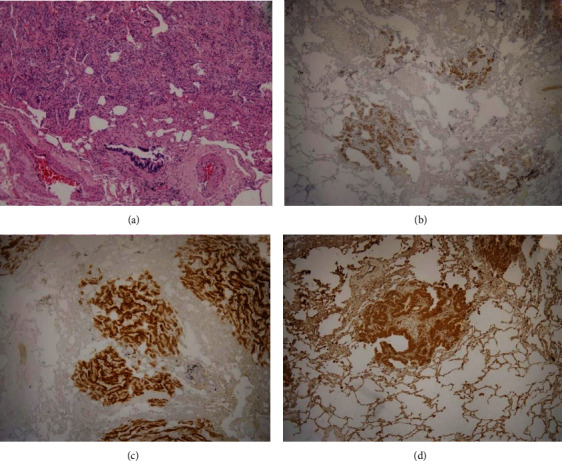
Lung parenchyma with meningothelial cells that are solid trabecularly arranged and has a round to ovoid shape; in some places, they are fusiform (hematoxylin and eosin, 100x) (a). Immunohistochemical staining was positive for progesterone receptor (b), CD56 (c), and vimentin (d).

A 60-year-old, smoking man was admitted to the pneumology department for the investigation of a chest X-ray abnormality that was found on a routine medical examination. His past medical history included previous *carotid artery* disease with *stroke*, arterial hypertension, nephrolithiasis, and urolithiasis. He did not have any known malignancy. The physical examination of the patient was within normal limits. Current high-resolution computed tomography (HRCT) of the thorax at the time of admittance displayed diffuse, multiple, 1-5 mm nodular lesions in the parenchyma of both lungs, with a slight predominance in the upper lobes (Figure 1). His laboratory test values and tumour marker levels were normal. Tuberculin skin test was negative. Bronchoscopy showed a normal endobronchial finding. The analysis of bronchoalveolar lavage fluid specimens showed no tumour cells, and the CD4/CD8 ratio in bronchoalveolar lavage fluid analyses via flow cytometry was 7.5. The examination of transbronchial biopsy specimens showed normal peribronchial lung parenchyma with a micronodule of benign neuroendocrine hyperplasia, without any signs of granulomatous inflammation. Pulmonary function test results revealed normal spirometry, lung volumes, and diffusion capacity. The patient was indicated for videothoracoscopic lung biopsy. The patient underwent right video-assisted thoracoscopic surgery (VATS) with atypical pulmonary resection of the upper lobe. Diffuse multiple nodules were observed on the visceral pleural surface during VATS. Histopathological examination revealed small nodules ranging from 1 to 5 mm in perivascular localization. Immunohistochemically, the tumour population expressed vimentin, CD56, and progesterone receptor and there was a negative expression of other neuroendocrine markers (synaptophysin, chromogranin A, and InsM1) and also negative for CK7, TTF-1, p40, CK5/6, HMB45, AE1/AE3, S100, and desmin (Figure 2). The proliferative activity (Ki-67) was very low. A rare PDM was diagnosed.

## 3. Discussion

In 1960, Korn and colleagues [1] first described pulmonary meningothelial nodules as “pulmonary tumours resembling chemodectomas.” As it was later shown, these tumours do not have neuroendocrine differentiation and are histologically similar to meningiomas [2]. Other terms used in literature are pulmonary meningothelial-like nodules, minute pulmonary meningothelial-like nodules (MPMN), pulmonary chemodectoma, and MPMN-omatosis syndrome [3].

The frequency of DPM in autopsy-based analyses ranges from 0.3% to 0.5%, and the frequency in operation-based analyses ranges from 1.1% to 9.5% [3–6]. DPM is more prevalent in females and more common in the 6th decade. The etiology of DPM is at present unclear. Some studies suggest that hormonal imbalance with progesterone stimulation, chronic lung diseases with inducing hypoxia, and ischemia may provide a cellular growth stimulation to DPM [2]. The authors, however, found out that the patients with DPM showed greater genetic instability with multiple losses of heterozygosity and they might represent transition between the reactive and neoplastic processes [4].

DPM is usually asymptomatic and tends to coexist with active pulmonary malignancy (lung adenocarcinoma), chronic ischemic heart disease, and pulmonary thromboembolism [3, 6].

Nodules are detected on high-resolution computed tomography as tiny nodules (2-5 mm in diameter) or ground-glass opacity accidentally. DPM can mimic the appearance of a wide spectrum of multiple lung nodule diseases on a chest-computed tomography scan. There are mainly three categories of disease: nonneoplastic, metastatic neoplasms from a known or an unknown primary malignancy, and rare primary lung neoplasms [7]. More common causes of multiple lung nodules displayed on CT are nonneoplastic conditions such as infections (military tuberculosis, fungal infection, and nontuberculous mycobacteriosis) and noninfectious lung diseases as sarcoidosis, necrotising sarcoid granulomatosis, hypersensitivity pneumonitis, hot tub lung, berylliosis, amyloidosis, granulomatosis with polyangiitis, eosinophilic granulomatosis with polyangiitis, rheumatoid nodules, talc granulomatosis, Langerhans cell histiocytosis, and bronchocentric granulomatosis [8]. DPM can mimic the appearance of hematogenous lung metastasis, primary adenocarcinoma of the lungs, and lymphangitis carcinomatosa. The rare neoplasms belong to the next category presented as multiple synchronous unilateral or bilateral lung indeterminate nodules on chest CT: primary pulmonary lymphomas (pulmonary extranodal marginal zone lymphoma of mucosa-associated lymphoid tissue, lymphomatoid granulomatosis, posttransplant lymphoproliferative disorder and diffuse large B-cell lymphoma), tumours of vascular origin (epithelioid haemangioendothelioma, angiosarcoma, Kaposi sarcoma), and neuroendocrine tumours (diffuse idiopathic pulmonary neuroendocrine cell hyperplasia) [7].

The differential diagnosis of patients presenting with diffuse nodules is very important; the multidisciplinary team approach is needed. Routine diagnostic work-up is a matter of course, and definitive diagnosis of DPM always requires histopathology. DPM have been diagnosed also by using transbronchial biopsy, but mostly by videothoracoscopic lung biopsy. It is a safe modality with a high rate of diagnostic success. Positron emission tomography imaging in the diagnosis of multiple lung nodules have a role in the evaluation of indeterminate lung nodules larger than 8 mm in patients with low-to-moderate probability of malignancy [9]. Smaller nodules like DPM may not be detected.

DPM consists of epithelioid cell nests or whorls in a zellballen-like arrangement centered on small veins [1, 4]. Histologically, the lesions were composed of small clusters of epithelioid cells with round to oval nuclei devoid of atypia and surrounded by abundant eosinophilic cytoplasm. Immunohistochemical studies show that cells comprising DPM stain positive for epithelial membrane antigen, vimentin, and progesterone receptor and negative for cytokeratin, actin, S-100 protein, CD34, chromogranin, and synaptophysin [10].

In therapeutical management of DPM, conservative treatment with observation and follow-up chest computed tomography imaging is usually favored [11].

## 4. Conclusion

This rare eventuality of DPM should be considered in patients with nonspecific diffuse bilateral pulmonary nodules on high-resolution computed tomography. The differential diagnosis of these patients is very important. DPM can mimic several serious lung malignancies, infection, and interstitial lung diseases. Definitive diagnosis of DPM requires histopathology and most often videothoracoscopic lung biopsy.

## Data Availability

Further details and information about the case are available upon request. The contact person is Anton Dzian (anton.dzian@jfmed.uniba.sk).
